# A Case of Oxalate Nephropathy Associated With Prolonged Cholecystostomy Tube Placement

**DOI:** 10.7759/cureus.40797

**Published:** 2023-06-22

**Authors:** Amanda G Hale, Derek S Anderson, Tara Eineichner, Christian M De Souza, Elias Smirlis, Babajide Adio

**Affiliations:** 1 Medicine, Des Moines University College of Osteopathic Medicine, Des Moines, USA; 2 Internal Medicine, University of North Dakota School of Medicine and Health Sciences, Des Moines, USA; 3 Internal Medicine, MercyOne North Iowa Medical Center, Mason City, USA

**Keywords:** renal sclerosis, cholecystectomy, bile salts, nephropathy, oxalate crystals

## Abstract

Oxalate nephropathy is a rare pathology that can be difficult to diagnose. It results from calcium oxalate crystals that are deposited in the renal interstitium or renal tubules. Once the deposition ensues, a multitude of complications can occur, including renal failure. One etiology for oxalate nephropathy is a lack of biliary acid. The diagnosis of oxalate nephropathy is typically based on visualization of oxalate crystals in the renal tubules on biopsy, and treatment based on the etiology can range from simple removal of the offending agent or a change in diet to liver/kidney transplant in the setting of primary hyperoxaluria. This report discusses a case of severe oxalate nephropathy related to long-term cholecystostomy tube placement resulting in a deficiency of biliary acid.

## Introduction

Oxalate nephropathy is a rare cause of kidney injury and its diagnosis is often missed [1,2]. It manifests as deposits of crystalline calcium oxalate causing interstitial fibrosis and tubular injury resulting in acute or chronic kidney disease [1-3]. There are two broad etiologic categories of oxalate nephropathy: primary oxalate nephropathy and secondary oxalate nephropathy [1-6]. Primary oxalate nephropathy arises from genetic inborn enzymatic errors in the glyoxylate pathway causing an overproduction of oxalate [1,2]. Secondary oxalate nephropathy has several etiologies, all of which can be attributed to a non-genetic increase in oxalate concentration within the kidney [1-6]. Increased ingestion of oxalate, associated with the ingestion of ethylene glycol ingestion or foods rich in oxalate or vitamin C, can lead to increased oxalate deposition within the kidney [1-3]. Oxalate-rich foods include leafy plants, black tea, star fruit, parsley, and nuts [2]. Additionally, fat malabsorption can lead to increased enteric absorption of oxalate. Common etiologies of fat malabsorption include gastric bypass, Crohn’s disease, and chronic pancreatitis [1-6]. Decreased oxalate excretion resulting from chronic renal disease and/or decreased water intake will also increase oxalate deposition within the kidneys [2-5]. Regardless, the accumulation of oxalate within the renal parenchyma can lead to more common pathologies such as nephrolithiasis, nephrocalcinosis, and the rarer oxalate nephropathy [1,2].

## Case presentation

An 87-year-old Caucasian female presented to the hospital after suffering a fall and her hospital course was complicated by acute kidney injury (AKI) on chronic kidney disease (CKD) stage IIIB. She denied any symptoms of a syncopal episode. However, she had been having difficulty ambulating a few days prior to the admission and was being treated for a urinary tract infection (UTI), yielding mixed Gram-positive bacteria on the day prior to the admission. Her relevant past medical history included recent acute cholecystitis managed with a percutaneous cholecystostomy five weeks prior to the admission, CKD stage IIIB with a baseline creatinine (Cr) of 1.3-1.7 mg/dL, atrial fibrillation, heart failure with preserved ejection fraction (HFpEF), diabetes mellitus type 2, obesity, hypertension, hyperlipidemia, and obstructive sleep apnea. On presentation, the patient’s blood urea nitrogen level was 130 mg/dL, and Cr was 11.39 mg/dL. Leukocytosis was present at 20.77 x 10^3^/μL with an elevated neutrophil percentage at 91.7%. Urinalysis was significant for +2 protein, +3 leukocyte esterase, +1 bacteria, and greater than 100 white blood cell (WBC) clumps, prompting reflexive urine culture prior to initiating ceftriaxone. Additionally, intravenous (IV) 0.9% saline was administered, and Nephrology was consulted. In the setting of cholecystostomy drain, an initial CT of the abdomen and pelvis and a chest X-ray were unremarkable. The urine culture was positive for Candida glabrata, and fluconazole was initiated.

**Figure 1:**
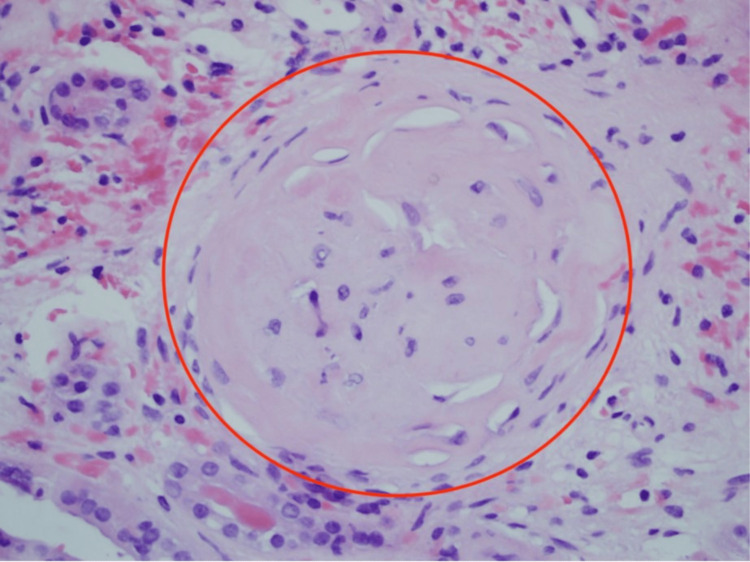
Extensive arteriosclerosis of right kidney – hematoxylin & eosin stain 400x magnification

**Figure 2:**
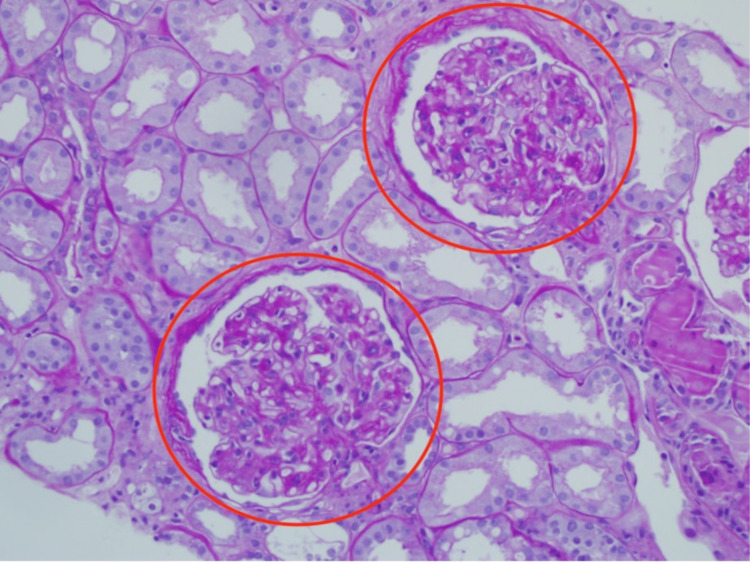
Extensive mesangial sclerosis of right kidney – hematoxylin & eosin stain 400x magnification

However, as the UTI was being treated, the patient’s renal function failed to improve despite resuscitation with IV fluids, resulting in oliguria. Due to the decline in renal function, Interventional Radiology was hesitant on removing her cholecystostomy drain, which had been scheduled for removal at six weeks from insertion. The patient’s mental status also started to decline with the most probable cause being uremic encephalopathy. In addition to the new-onset encephalopathy, she was found to be fecally incontinent with diarrhea. Nephrology conducted a diuretic challenge with 100 mg furosemide and 250 mg chlorthalidone, which failed, and dialysis was started on hospital day five. While renal function remained stable, her mental status did not return to baseline following dialysis. An electroencephalogram (EEG) demonstrated toxic metabolic encephalopathy. Levetiracetam was started and mental status steadily improved with waxing and waning of clarity throughout the day. A kidney biopsy was obtained for further AKI workup, which revealed acute oxalate nephropathy, diffuse diabetic glomerulosclerosis class IIa, and severe hypertensive arteriosclerosis. The findings of the renal biopsy raised concern for exacerbation of oxalate nephropathy in the setting of significant underlying chronic disease. Biopsy of the kidney under light microscopy demonstrated glomerular enlargement with mild to moderate mesangial sclerosis along with luminal ectasia, epithelial simplification, and enlarged nuclei with hyperchromasia in tubules and interstitium as shown in Figures 1-2.

**Figure 3:**
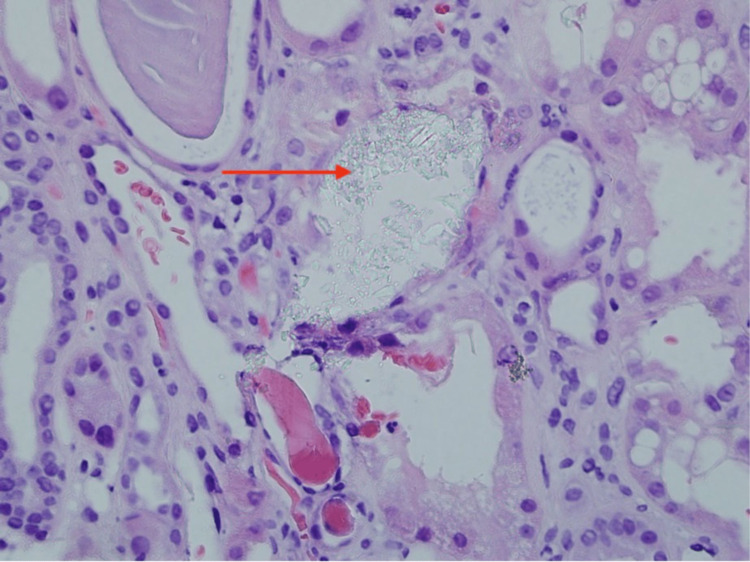
Calcium oxalate crystals in the tubular lamina of right kidney – hematoxylin & eosin stain 400x magnification

Arteriosclerosis and arteriolar hyalinosis were present in vessels with abundant calcium oxalate crystals in the tubular lamina, as seen in Figure 3.

Oxalate nephropathy and subsequent acute renal failure in the patient likely resulted from a combination of risk factors including chronic underlying diabetic and hypertensive glomerular disease as well as the cholecystostomy tube. The patient was ultimately discharged to at-home hospice care.

## Discussion

Oxalate nephropathy is a difficult diagnosis to make as it is a rare entity that has been estimated to be the cause of kidney disease in just 1% of native kidney biopsies [7]. This condition can be diagnosed either via pathologic visualization of oxalate crystals within the renal tubules [2] or via 24-hour urine oxalate measurements; however, "it is not common practice" to collect 24-hour urine oxalate measurements outside of the setting of nephrolithiasis [1-2,4-5]. The differential can be expanded to include oxalate nephropathy by addressing steatorrhea or changes in diet that includes ingestion of foods rich in oxalate as well as the patient's surgical history, especially bowel surgeries [1-3,8].

A point of interest in this case report is the effect of bile acids on the absorption of oxalate. The effect of bile on oxalate absorption has been well described, albeit pertaining to the inability to reabsorb bile salts following gastric bypass or ileal dysfunction rather than an inability to secrete bile acid following cholecystostomy [1-6]. The pathway to oxalate nephropathy by means of bile acid loss is characterized by the loss of bile salts through the colon [1-6]. The loss of bile in stool causes an increase in the production of bile by the liver, although this increase in production is eventually overwhelmed leading to increased free fatty acids within the colon. Under healthy conditions, calcium binds to oxalate and is excreted in stool, but when calcium is bound to free fatty acids, oxalate is free to be enterically absorbed [2-3,6]. Additionally, the presence of bile acids within the colon increases colonic wall permeability to oxalate [3,6], which provides another mechanism for inappropriate oxalate absorption. In the case of our patient, an actively draining cholecystostomy had been present for five weeks. The absence of bile salts would cause fat malabsorption, which constitutes one piece of the enteric hyperoxaluria puzzle. This was likely the etiology of oxalate nephropathy in our patient; however, we have been unable to find any prior studies or case reports on this particular pathophysiology.

The treatment options for acute oxalate nephropathy vary and are based on the etiology. In primary hyperoxaluria, the treatment aims to modify the enzymatic pathways causing the increase in oxalate production [2]. For secondary hyperoxaluria, particularly enteric hyperoxaluria, the treatment primarily involves reversing the cause of increased oxalate absorption [2-3,5]. For example, if the patient had been on a high-fat and low-calcium diet leading to an increase in oxalate absorption, the initial treatment objective would be to increase calcium and fat intake to decrease oxalate absorption [2-3,5]. In our patient, the likely etiology of increased oxalate absorption was the continuously draining cholecystostomy, and hence the treatment in such cases would start with the removal of the drain. Several case series have provided evidence to support the increased risk of oxalate nephropathy among patients with hypertension and diabetes. One case series published in 2008 reviewed 11 patients who had undergone Roux-en-Y gastric bypass; in this series, all patients had hypertension and the majority had diabetes and chronic kidney disease [9]. In a series involving 12 patients with chronic pancreatitis, 67% of the patients had hypertension, 75% had diabetes, and one patient had undergone a renal transplant for diabetic nephropathy [10]. Buysschaert et al. [7] identified 22 cases of oxalate nephropathy from a Belgian database; this case series showed that 57% of patients were diabetic, 76% had hypertension, and 62% had underlying CKD with eGFR of 36 mL/min/1.73 m^2^ prior to the onset of oxalate nephropathy. Finally, in a case series involving 25 patients with oxalate nephropathy in the New York City area, 76% had hypertension and 64% had diabetes [8].

## Conclusions

Oxalate nephropathy is a rare pathologic disease characterized by increased deposition of oxalate within the renal parenchyma. Careful attention needs to be paid regarding recent diet and stool changes during history gathering, as well as past medical history related to the enterobiliary system. Additionally, in cases with high suspicion of oxalate nephropathy, a 24-hour oxalate measurement or renal biopsy can help confirm the diagnosis, which could enable prompt treatment without further renal injury; however, the gold standard for diagnosis is renal biopsy. Treatment options vary; however, in cases such as our patient, drain removal would be a good starting point to reduce baseline oxalate absorption.
